# Replicate Aptima Assay for Quantifying Residual Plasma Viremia in Individuals on Antiretroviral Therapy

**DOI:** 10.1128/JCM.01400-20

**Published:** 2020-11-18

**Authors:** Sonia Bakkour, Xutao Deng, Peter Bacchetti, Eduard Grebe, Leilani Montalvo, Andrew Worlock, Mars Stone, Steven G. Deeks, Douglas D. Richman, Michael P. Busch

**Affiliations:** a Vitalant Research Institute, San Francisco, California, USA; b Department of Laboratory Medicine, University of California San Francisco, San Francisco, California, USA; c Department of Epidemiology and Biostatistics, University of California San Francisco, San Francisco, California, USA; d Hologic, Inc., San Diego, California, USA; e Department of Medicine, University of California San Francisco School of Medicine, San Francisco, California, USA; f Veterans Affairs San Diego Healthcare System and University of California San Diego, La Jolla, California, USA; Rhode Island Hospital

**Keywords:** latent reservoir, quantification, residual RNA, ultrasensitive viral load, viremia, human immunodeficiency virus

## Abstract

Detection of residual plasma viremia in antiretroviral therapy (ART)-suppressed HIV-infected individuals is critical for characterizing the latent reservoir and evaluating the impact of cure interventions. Ultracentrifugation-based single-copy assays are sensitive but labor intensive. Fully automated replicate testing using a standard clinical viral load assay was evaluated as a high-throughput alternative for the quantification of low-level viremia. Four plasma samples from blood donors with acute HIV-1 infection and one viral culture supernatant were serially diluted into 25-ml samples to nominal viral loads ranging from 39 to <0.5 copies (cp)/ml. Each dilution was tested with 45 replicates (reps) using 0.5 ml/rep with the Aptima HIV-1 Quant assay. The nominal and estimated viral loads based on the single-hit Poisson model were compared, and a hybrid Poisson digital model for calibrated viral load estimation was derived. Testing performed using 45 reps on longitudinal plasma samples from 50 ART-suppressed individuals in the Reservoir Assay Validation and Evaluation Network (RAVEN) study cohort (range of 1 to 19 years of continuous ART suppression) showed a median viral load of 0.54 cp/ml (interquartile range [IQR], 0.22 to 1.46 cp/ml) and a 14% (95% confidence interval [CI], 9% to 19%) decline in viral load for each additional year in duration suppressed. Within the RAVEN cohort, the expected false-negative rate for detection at lower rep numbers using 9 and 18 reps was 26% and 14%, respectively. Residual plasma viremia levels positively correlated with cell-associated HIV RNA and DNA. The performance characteristics of the replicate Aptima assay support its use for quantifying residual plasma viremia to study the latent HIV reservoir and cure interventions.

## Abstract (precis)

Detection of residual plasma viremia in antiretroviral therapy (ART)-suppressed HIV-infected individuals is critical for characterizing the latent reservoir and evaluating the impact of cure interventions. Ultracentrifugation-based single-copy assays are sensitive but labor intensive. Fully automated replicate testing using a standard clinical viral load assay was evaluated as a high-throughput alternative for the quantification of low-level viremia. Four plasma samples from blood donors with acute HIV-1 infection and one viral culture supernatant were serially diluted into 25-ml samples to nominal viral loads ranging from 39 to <0.

## INTRODUCTION

Quantification of residual low-level viremia in research participants on antiretroviral therapy (ART) and during curative interventions requires ultrasensitive plasma HIV RNA assays that can measure viral loads below the limits of quantification or detection of the standard clinical viral load assays used for patient monitoring (now usually around 20 to 30 copies HIV RNA/ml plasma). The level of residual plasma viremia in participants on suppressive ART may reflect the size of the replication-competent latent reservoir (measured using viral outgrowth or molecular assays), the size of the expressed reservoir (based on cell-associated HIV RNA), and the number of HIV-infected cells (based on cell-associated HIV DNA). Associations have been observed between the levels or presence of residual plasma viremia and cell-associated virological markers (1–3), suggesting that residual viremia is a measure of virus production by latently infected cells that have become activated, although conflicting evidence has also been reported showing no association between residual viremia and *ex vivo* cell-based HIV measurements (4, 5). Importantly, detectable residual viremia was shown to be associated with shorter time to viral rebound upon treatment interruption (3). Thus, tracking plasma residual viremia in cure studies provides insight into the effect of an intervention on virus eradication at the systemic level.

Ultrasensitive plasma HIV RNA assays should perform with reasonable sensitivity, specificity, precision, and dynamic range in order to be able to measure potentially small within-person changes or relative differences between study subgroups in residual viremia linked to observational or interventional studies for HIV cure research. Effectively treated participants on suppressive ART typically have low-level viremia that is only detectable with HIV-1 RNA assays with single-copy sensitivity (6, 7), and the ability to quantify a low copy number is largely driven by the volume of plasma assayed, usually ≥3 ml. Given that single-copy assays measure rare target entities in plasma, sampling variation is an issue that can be reduced with higher input volume, lowering the frequency of “undetected” results, meaning that no copies were present in the sample assayed (8). Several interventional studies within the last decade have measured within-person changes in residual viremia from before to after treatment, using single-copy assays based on ultracentrifugation of ∼3 to 7 ml of plasma (9–13). Viremia ranged from undetected to 23 copies/ml (cp/ml) across individuals, although the median viremia ranged from approximately 0.2 to 0.7 cp/ml.

In contrast to single-copy assays that rely on ultracentrifugation and lab-developed real-time reverse transcriptase PCR (RT-PCR), replicate tests can be performed on FDA-approved fully automated commercial nucleic acid amplification assays to measure low-level viremia in suppressed individuals. A limited number of studies have adopted this approach to track plasma HIV RNA levels in elite controllers before and after ART (14, 15). Up to 2 ml of plasma was assayed in quadruplicate testing, with a lower detection limit of <3 cp/ml, and quantification was based on comparing the average signal/cutoff (S/Co) ratio assay results, which ranged from 0 to 30, with S/Co of <1.0 classified as HIV RNA “negative” and S/Co of ≥1.0 classified as HIV RNA “positive.” Using the average S/Co ratio of quadruplicate tests to measure low-level plasma viremia, 45% of plasma samples from ART-suppressed individuals were undetected (15).

Many combination clinical trials are under way and may involve multiple concurrent or successive strategies either to induce latency reversal or to induce immune-based antiviral responses. Several studies aiming to induce latency reversal have relied on commercial assays to report increases in quantifiable viral load or probability of detectable viremia after treatment (16–18). Plasma HIV RNA levels may fluctuate above and below the limit of quantitation of standard commercial viral load assays, making results more difficult to interpret. An ideal plasma HIV RNA assay would thus have high sensitivity and a wide dynamic range, capturing both increases in plasma viremia resulting from latency reversal and decreases due to the reduction of the inducible latent reservoir as a step in an eradication strategy. While current ultracentrifugation-based assays can measure small amounts of HIV RNA pelleted from a large volume, they are limited in throughput, may not efficiently amplify diverse HIV sequences due to primer/probe mismatches, and may be susceptible to contamination and variability due to multiple parallel manual processing steps of samples and positive controls, resulting in nonspecific or imprecise results.

We investigated whether replicate (rep) testing using a commercial widely available viral load assay on a fully automated processing platform could allow high-throughput quantification of low-level viremia with possible benefits of precision, reproducibility, and ease of use.

## MATERIALS AND METHODS

### HIV-1 RNA assay.

The commercially available Aptima HIV-1 Quant Assay uses isothermal transcription-mediated amplification combined with specific target capture to quantitate HIV-1 from 10,000,000 cp/ml to 30 cp/ml (19, 20). It is performed on a fully automated platform (Hologic Panther) using 0.5-ml plasma samples. The Aptima HIV-1 Quant assay includes dual-target capture, amplification, and detection systems, targeting pol and LTR independently to ensure detection of diverse HIV-1 groups and subtypes (21, 22). The concentration of HIV-1 in a sample is calculated using the time taken for the fluorescent signal of each target to reach a specified threshold (tTime). The Panther software compares sample results to a calibration curve to generate the reported result.

The Panther platform reports HIV-1 concentrations between 30 and 10,000,000 cp/ml. Concentrations below 30 cp/ml may still generate a signal using the Aptima HIV-1 assay that is reported as “<30 detected” by the instrument. This signal can be used to estimate the level of HIV-1 below 30 cp/ml by extrapolation using proprietary Hologic software.

Another approach to quantify lower-level viremia is to use replicate testing and measure the number of reps that give a positive result versus the total number of reps tested. This approach has the advantage that it can be done using the Panther reported results without the need for extrapolation, although at least one negative replicate is required. For each replicate, the commercial instrument software returns three possible results, as follows: a quantitative concentration of ≥30 cp/ml, a detected signal (<30 cp/ml) that is not quantified by the instrument software, or an undetected signal. The Panther instrument can be programmed to generate up to 9 distinct reps for each 5-ml tube of plasma loaded onto the instrument, with the option of loading multiple 5-ml aliquots to further enhance sensitivity with additional reps. Each rep can be classified as positive or negative based off the reported results from the Panther instrument. This classification can easily be translated into a positivity rate for each sample (positive reps/total number of reps tested). Poisson model calculations can then be used to estimate the cp/ml (23).

### Serial dilution “calibration” samples.

Initially, a subtype B HIV-1 inactivated viral culture supernatant (NIBSC code 10/152) with a low viral load working stock of 195 (95% confidence interval [CI], 184 to 207) cp/ml (measured using 90 reps of the Aptima assay) was serially diluted 2-fold into defibrinated plasma (Seracare) to generate 6 nominal concentrations ranging from 98 to 3 cp/ml. Each dilution was tested using 90 reps (5 reps per aliquot × 18 aliquots), resulting in 54% of reps detected at the lowest concentration. To further extend the testing range, the 3-cp/ml dilution was further diluted 2-fold with the same lot of defibrinated plasma to generate 4 additional lower nominal concentrations ranging from 1.5 to 0.2 cp/ml. Each of the additional dilutions was tested in 38 to 49 reps (up to 8 reps per aliquot × 5 to 7 aliquots). To mimic diverse clinical samples from controllers and ART-suppressed individuals, four plasma samples from blood donors with acute (RNA positive/antibody negative) HIV-1 infection (two subtype B and two subtype C) were also serially diluted in defibrinated plasma. The viral loads of the working stocks for each sample (measured using the Aptima assay in 4 or 13 reps) were 281 (95% CI, 264 to 299) cp/ml, 16 (95% CI, 11 to 24) cp/ml, 97 (95% CI, 87 to 109) cp/ml, and 291 (95% CI, 261 to 324) cp/ml for B-1, B-2, C-1, and C-2, respectively. Each sample was serially diluted 3-fold to generate 5 concentrations, with the lowest nominal concentration of <0.5 cp/ml. Each dilution series was tested in a blind manner in 45 reps (9 reps per aliquot × 5 aliquots).

### Clinical samples.

The Reservoir Assay Validation and Evaluation Network (RAVEN) study cohort includes HIV-infected individuals taking ART for at least 1 year with HIV RNA plasma viral loads of <40 cp/ml, categorized as having initiated ART during “early” or “chronic” infection based on beginning ART <6.5 months or >12 months since the estimated date of HIV infection, as well as HIV-negative individuals. Participants in the RAVEN project were enrolled and followed as part of the University of California San Francisco (UCSF) Options and SCOPE programs, with specific consent for apheresis collections and testing for this study as approved by the UCSF Committee on Human Research (institutional review board [IRB]) number 10-03244. The study population consisted of 50 HIV-infected individuals (25 early and 25 chronic ART initiation; range, 1 to 19 years of continuous ART suppression), which were all infected with HIV-1 clade B, and 10 HIV-negative individuals. During apheresis, 80 ml of mononuclear cells (MNCs) and 250 ml plasma were collected over the course of about 2 hours on the Spectra Optia device using the continuous MNC collection program, wherein the MNC product is continuously collected from whole blood that is cycled into the device and collected into the collection bag, followed by reinfusion of all uncollected products. Plasma containing an anticoagulant citrate dextrose solution A (ACD-A) anticoagulant was collected last after the MNC product had been collected. Participants averaged about 1 to 1.5 total blood volume processed by the device. Apheresis-derived samples were collected and cryopreserved from each participant, ranging from 1 to 6 visits spanning up to 2 years. A total of 104 plasma samples from HIV-infected participants and 10 samples from HIV-negative individuals were tested in 45 reps each, with testing performed in a blind manner for each sample on five independently coded 5-ml aliquots (9 reps per aliquot), with the exception of 2 samples from HIV-infected participants, for which testing was performed in 18 or 27 reps. CD4^+^ T cells were isolated from peripheral blood mononuclear cells (PBMCs), and nucleic acid (genomic DNA and RNA) was extracted using the Allprep DNA/RNA/miRNA universal kit (Qiagen). Cell-associated HIV RNA, total DNA, and integrated DNA were quantified by quantitative PCR (qPCR) in 3 replicates each of the extracted nucleic acid and normalized to cell equivalents using human genomic RPLP0 expression (for RNA) or CD3 (for DNA) (24, 25).

### Statistical analyses.

**(i) Derivation of viral load estimate.** For each of the 5 calibration samples tested in serial dilutions, the viral load estimate was calculated for the highest concentration using an open access statistical tool for limiting dilution assays (23). This calculator produces maximum likelihood estimates for viral load (VL), which were based on the single-hit Poisson model using inputs from the total number of reps and number of positive reps at each dilution. In addition to providing a maximum likelihood estimate, this calculator returns a 95% confidence interval.

**(ii) False-negative rate.** A false-negative rate is defined as the probability that all reps are negative at certain concentrations when HIV RNA is present in the specimen based on enhanced testing. Theoretically, at 1 cp/ml, an assay with perfect sensitivity with 9 reps (rep volume, 0.5 ml) has a 1.1% chance to report all negative reps, which occurs only when the 4.5 ml assayed happens to contain no copies due to Poisson sampling variation. Using 18 reps has a 0.012% chance to report negative results, a 90-fold improvement over the 9-rep assay, assuming single-copy sensitivity. For each concentration, the false-negative rate was calculated as the probability that a Poisson variable will equal zero when its mean is equal to cp/ml times ml assayed. For our assay which is less sensitive than the perfect assay, the above false-negative rate for 9 reps would correspond to a true concentration of 1.6 cp/ml.

The VL estimates from the 50 RAVEN cohort participants at study entry were analyzed to calculate the expected number of false negatives for this cohort at 9, 18, and 45 reps. Five participants were excluded from the analysis due to no detection using the Aptima assay in all 45 reps, and 1 participant was excluded from the analysis due to VL above the lower limit of quantitation of the standard Aptima assay (in a single rep). The false-negative probability for each of the remaining 44 participants at a given number of reps was calculated based on their estimated VL. The expected number of false negatives for this cohort was calculated by summing those false-negative probabilities for the 44 participants.

**(iii) Viral load declines over time.** The replicate testing results from the RAVEN cohort were analyzed to estimate the average decline in viral load and the potential impact of early versus chronic ART initiation on the rate of decline. We modeled the viral load decline using negative binomial mixed effects regression using a log link with “effectiveCopies” as the outcome, “effectiveVolume” as the exposure (offset), and duration of suppression as the predictor. We estimated a population average rate of decline (fixed effect) and subject-specific (random) intercepts. Timing of ART initiation (chronic versus early) was not statistically significant when added to the model and therefore was not included in the final model. One early participant (with 2 visits) was excluded from the analysis due to a brief period of treatment interruption prior to the RAVEN study. One chronic participant (with 1 visit) and 1 of 2 visits from an early participant were excluded from analysis, due to detection using the Aptima assay in all 45 reps. When all reps are positive, the Poisson-based replicate viral load estimation method is not applicable. In total, 100 longitudinal samples from 48 participants were included in the analysis.

The adjusted intraclass correlation coefficient, which measures the between-person variance as a proportion of total (within plus between) variance, was estimated using random-intercept-random-slope negative binomial mixed effects regression with effectiveCopies as the outcome, effectiveVolume as the exposure, and time in study as the predictor.

**(iv) Correlation of plasma viral load and cell-associated HIV reservoir parameters.** We evaluated the correlation between plasma viral load and cell-associated HIV RNA and DNA using negative binomial generalized estimating equation models that account for intrasubject correlation. In total, data were available from 89 longitudinal samples from 49 participants. We also modeled changes in cell-associated HIV RNA and DNA over time, using a negative binomial model similar to that used for viral load decline, with HIV copies as the outcome and cell equivalents as exposure.

## RESULTS

### Quantification of low-level viremia based on replicate readouts.

**FIG 1:**
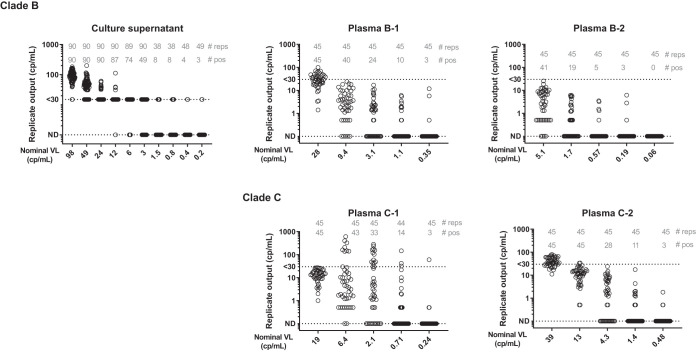
Replicate Aptima testing results for serially diluted HIV culture supernatant and donor plasma samples. For each nominal viral load in cp/ml (on the *x* axis), the total number of replicates and the number of positive replicates are shown at the top of each graph, as well as the individual replicate results in cp/ml (on the *y* axis). ND, not detected.

Serially diluted HIV-positive samples were tested with the Aptima HIV Quant assay using a median of 45 reps (range, 38 to 90 reps). The serially diluted viral culture supernatant sample with a nominal concentration of 12 cp/ml was detected as positive in 87/90 (97%) of reps, whereas at a nominal concentration of 0.2 cp/ml, 3/49 (6%) of reps were positive (Fig. 1). Likewise, the serially diluted donor plasma samples with nominal concentrations of 4 to 9 cp/ml were detected as positive in 62% to 96% of 45 reps, whereas at the lowest nominal concentrations of 0.2 to 0.5 cp/ml, they were detected as positive in 3/45 (7%) of reps.

**FIG 2:**
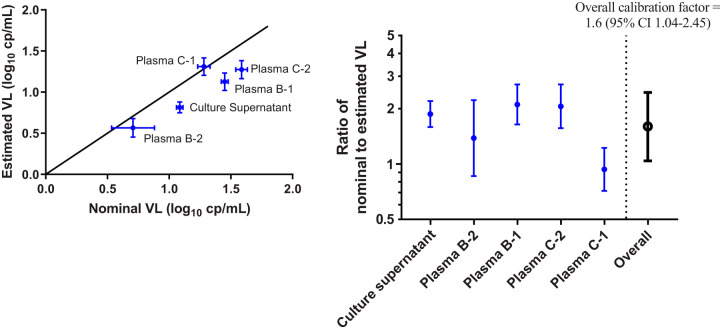
Comparison of nominal and estimated viral load based on the single-hit Poisson model. Data for each of the 5 individual standards are shown in blue. The overall calibration factor (black open circle) was calculated as the geometric mean of the 5 individual standard calibration factors. Error bars, 95% confidence interval.

Several strategies for low viral load estimation were evaluated (see Appendix SA in the supplemental material), based on using quantitative (i.e., extrapolated concentrations of <30 copies/ml) or digital (i.e., each replicate classified as positive or negative) methods to estimate copy number from replicate testing results of the serially diluted HIV-positive samples. By comparing the estimated copy number for each method to the nominal concentration, we found that a hybrid Poisson digital model produced the best correlation and accuracy (see Appendix SA and Tables SA1 and SA2 in the supplemental material). While VL estimates can be calculated from the positive frequency of replicates by application of Poisson distribution analysis, the latter does not account for imperfect assay sensitivity. Indeed, viral load estimates based on the single-hit Poisson model for each of the five serially diluted samples were on average 0.2 log_10_ (range, 0 to 0.3 log_10_) lower than the nominal concentrations (Fig. 2). To calibrate the VL for each sample, a calibration factor was calculated as the ratio of the nominal VL to the maximum likelihood estimate. The overall calibration factor was derived as the geometric mean of the 5 separate calibration factors. In order to obtain a confidence interval for the overall calibration factor, we used the between-sample standard deviation (SD) of the calibration factors on the log scale to estimate the confidence interval for the overall calibration factor. Thus, Poisson model-based VL estimates derived from digital readouts were multiplied by a calibration factor of 1.6 for the quantification of low-level viremia.

A spreadsheet (see Appendix SB in the supplemental material) with the calibrated estimates of cp/ml and their CIs was created using a SAS program, based on all possible combinations of the number of reps (up to 45) and how many of those are positive, assuming a calibration factor of 1.6. The CIs reflect both the uncertainty in the maximum likelihood estimates and variance of the calibration factor. The program also created “stand-in” data such that a Poisson regression fit to the single observation of effectiveCopies with exposure variable effectiveVolume (included as an offset) will reproduce the cp/ml and its CI. The equivalent copies and volume provide a way to analyze a study’s VL results from digital Panther assays using regression methods for count data (8) in a way that properly reflects the VL results’ precision.

### Performance of replicate Aptima assay at various numbers of replicates.

**FIG 3:**
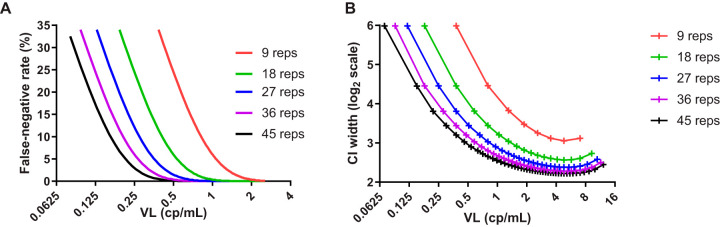
Modeling the effect of lower replicate numbers on the false-negative rate of detection (A) and precision of the viral load estimate (B).

The estimated VL is 0.38 cp/ml when 1 positive rep is detected out of 9 reps tested, and the VL is 0.07 cp/ml when 1 positive rep is detected out of 45 reps tested. Since limited plasma volumes (5 to 10 ml) may be available for testing samples from clinical trials, the effect of lower rep testing on false-negative rates was modeled. For a viral load of 1 cp/ml, the expected false-negative rate for detection using 9 reps and 18 reps was 6% and 0.3%, respectively, whereas for a viral load of 0.5 cp/ml, the expected false-negative rate rose to 25% and 6% for 9 reps and 18 reps, respectively (Fig. 3A).

The effect of lower rep testing on the precision of the VL estimate was also modeled. Relative precision, as reflected in the CI width around the logarithm of the VL estimate, was modeled for multiples of 9 reps, up to 45 reps, across the range of possible Poisson model-based VL estimates. The highest relative precision (i.e., lowest CI width for the estimate of log VL) was achieved at a VL of 5 cp/ml, wherein testing with 18 reps relative to 9 reps decreased the width of the confidence interval around the log VL estimate by 29%, whereas incrementally increasing the reps beyond 18 gave smaller gains in log-scale precision by 12%, 6%, and 4% as the number of reps increased to 27, 36, and 45, respectively (Fig. 3B). At a VL of 0.8 cp/ml, testing with 18 reps relative to 9 reps decreased the width of the confidence interval around the log VL estimate by 50%, while each additional increment decreased the width of the confidence interval by 27%, 13%, and 10% at rep numbers of 27, 36, and 45, respectively.

To empirically derive performance estimates, 18 or 9 reps were randomly drawn from the 45 reps performed on the donor plasma samples to test the robustness of the method in a smaller sample size (Appendix SA). Spearman correlation coefficients of the estimated copy numbers relative to the nominal concentrations ranged from 0.95 to 0.99 and from 0.91 to 0.98 for 18 and 9 reps, respectively. The fold difference between the estimated and nominal concentrations when 18 and 9 reps were randomly sampled ranged from 1.2 to 1.7 and from 1.2 to 2.0, respectively.

### Detection of residual viremia in ART-suppressed individuals using replicate Aptima assay.

**FIG 4:**
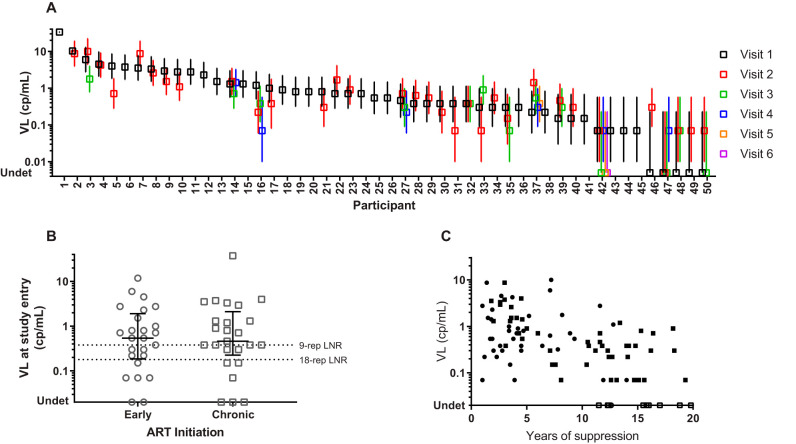
Application of the replicate Aptima assay (45 reps) to the RAVEN cohort of 50 ART-suppressed individuals with 1 to 6 collections within a 2-year period (A), stratified by timing of ART initiation (B), and in longitudinal samples as a function of years of continuous ART suppression (C). Viral load estimates are indicated by symbols in panel A, with error bars indicating upper and lower confidence intervals around the estimate. Lines and error bars in panel B indicate median VL and IQR, respectively. LNR, the lowest nonzero result. Circles and squares in panel C indicate ART initiation during early and chronic infection, respectively. Open symbols in panel C indicate undetectable VL.

Residual viremia in 50 ART-suppressed and 10 HIV-negative individuals was measured using 45 reps of the Aptima assay. No detection was observed in 447 reps (researchers were blind to HIV-negative control samples), with valid results from 10 HIV-negative individuals. Five of the 50 ART-suppressed individuals had undetectable VL upon study entry; however, in at least 1 longitudinal follow-up sample, at least 1 positive rep was detected out of 45 reps (Fig. 4A). Viral loads were reasonably stable within individuals over the study period. The intraclass correlation coefficient, which measures the between-person variance as a proportion of total (within plus between) variance, was 0.60. The confidence intervals around log(VL) widened with decreasing viral loads.

In ART-suppressed individuals in the RAVEN cohort, the median number of positive reps was 7 out of 45, corresponding to a viral load of 0.54 cp/ml (interquartile range [IQR], 0.22 to 1.46 cp/ml) (Fig. 4B). Viral loads were similar between individuals who initiated ART during early versus chronic infection. Two ART-suppressed individuals in the RAVEN cohort had all positive reps at study entry, although they differed in that one had a level of viremia (37 cp/ml) that was within the quantifiable range of the standard Aptima assay (in a single rep), whereas the other had viremia (12 cp/ml) that was detectable but below the limit of quantitation of the assay, requiring proprietary extrapolation analysis (Hologic) to derive the VL based on the average of all extrapolated low-level viral load results. At study entry, the expected number of false negatives for the ART-suppressed individuals in the RAVEN cohort at rep numbers of 9, 18, and 45 was 11.5 (26%), 6.3 (14%), and 2.2 (5.0%), respectively.

For 48 ART-suppressed individuals, viral loads were analyzed in samples collected at a single time point or longitudinally over the course of up to 2 years, after a period of continuous ART suppression ranging from 1 to 19 years (Fig. 4C). Across individuals, for each 1-year increase in duration suppressed, VL decreased by 14% (95% CI, 9% to 19%). Timing of treatment initiation (early versus chronic treated) was not a statistically significant predictor of plasma VL, but time suppressed remained statistically significant. Quantitative levels of cell-associated HIV RNA, total DNA, and integrated DNA were statistically significantly associated with plasma VL. For each additional 1-cp/ml plasma VL, the cell-associated HIV RNA copies/10^6^ cells was 6% (95% CI, 1% to 11%) higher, total DNA was 7% (95% CI, 1% to 12%) higher, and integrated DNA was 5% (95% CI, 0% to 11%) higher. Time suppressed was not a statistically significant predictor of cell-associated HIV RNA or DNA.

## DISCUSSION

Low-level viremia can be quantified based on reactive/nonreactive digital assay readouts on multiple 0.5-ml plasma reps of the Aptima assay with Poisson analysis, using a correction factor that accounts for imperfect sensitivity. Rather than a theoretical 100% detection of 1 copy/0.5-ml plasma input/aliquot, we estimated that each single copy has a 62.5% chance of yielding a reactive result. Viremia can be (and in this study was) detected in all individuals on long-term ART, although most have a VL of <1 cp/ml. A previous study (6) has estimated that viremia levels exhibit first- and second-phase decay rates, with half-lives of 1.5 and 28 days, respectively, during the first 5 weeks post-ART, followed by third- and fourth-phase decay rates, with half-lives of 39 weeks and infinite, respectively, between weeks 60 and 384 (1 to 7 years) post-ART. A subsequent study of ART-suppressed individuals with a longer-term follow-up (7) estimated that, after 4 years of ART, viremia decays 6% per year, with an estimated half-life of 11.5 years. A recent study measured 15.7% per year decay of intact proviral DNA in HIV-1-infected individuals from initial suppression through 7 years on suppressive ART (26). Likewise, in our study, despite VL appearing stable over the study period within individuals, we see clear evidence of a VL decline over time suppressed at the population level (14% per year decline).

The Aptima HIV-1 Quant viral load assay is widely available and the Panther platform has the unique capacity to generate 9 independent reps from a single 5-ml loaded sample. The replicate Aptima assay reported here has been applied to pilot studies to detect residual viremia in cases of HIV-infected cancer patients undergoing monoclonal antibody and drug therapy for targets thought to modulate the HIV reservoir (27–29). These studies used relatively fewer reps (9 to 18 reps) than reported in the current study, but this difference had only a small impact on the rates of detection and estimated viral loads linked to interventions. Furthermore, Jacobs et al. evaluated the replicate Aptima assay using 9 reps in comparison to the manual single-copy assay targeting *integrase* (iSCA v2), showing comparable sensitivity (30, 31).

In this study, residual plasma viremia levels positively correlated with cell-associated HIV RNA and DNA, suggesting that low-level plasma HIV RNA reflects the active reservoir at the systemic level. Characterization of the RAVEN cohort plasma samples using 45 reps of the Aptima assay detected plasma viremia in all 50 ART-suppressed participants and enabled us to pedigree the RAVEN sample repository using large volumes of plasma (25 ml). Thus, the highly pedigreed samples in the RAVEN repository, which are also characterized by multiple cell-associated molecular assays and quantitative viral outgrowth assays of PBMCs and CD4 cells, can be used as a resource when validating and evaluating novel reservoir assays.

However, this volume of plasma and number of reps yielded a sensitivity and precision level that are difficult to routinely achieve. Notably, the majority of participants in a typical highly suppressed cohort, with a viral load distribution in the range of 0.5 to 1 copy/ml, will have a low viral load within the Poisson estimation range. In the RAVEN cohort, 2% (1 out of 50) participants had all positive reps and viral loads in the 12- to 30-cp/ml range, which would require proprietary extrapolation analysis on a single rep. Since extrapolation analysis may not be scalable, a development of approaches is needed to measure viral load in the range where all reps may be positive and therefore above the Poisson model-based estimation but below the range of quantitation of the assay. One approach might be to initially test all samples using a single 0.5-ml rep. If the single rep is quantifiable, no further testing is required, while if the result is detected but below quantitation levels, a dilution (such as 1:3) should allow the viral load to fall within the 9-rep Poisson estimation range without risking a false-negative result. A nonreactive initial rep would be followed by 9-rep Poisson estimation without any dilution. However, this type of approach has limitations based on the branching strategy. Based on the particular design of the study, the number of reps chosen should consider the desired lowest nonzero result, precision, and false-negative rate, in addition to the practical considerations of available sample volume and cost.

In this study, we demonstrated that measurement of residual plasma viremia using the replicate Aptima assay shows a positive correlation with cell-associated HIV DNA and RNA levels, as well as a negative correlation with length of suppression, indicating that low-level plasma viremia reflects the systemic HIV reservoir. This assay could be particularly valuable for tracking the effect of latency reversal agents in pilot cure trials and may be used for detecting early viral rebound following treatment interruption.

## Supplementary Material

Supplemental file 1

Supplemental file 2
