# Supplementary material for: Replicate Aptima Assay for Quantifying Residual Plasma Viremia in Individuals on Antiretroviral Therapy
Source: J Clin Microbiol. 2020 Nov 18;58(12):e01400-20. doi: 10.1128/JCM.01400-20 (PMC7685884; doi:10.1128/JCM.01400-20)

Appendix A: Comparison of different strategies for low viral load estimation

Replicate testing data (45 replicates per dilution × 5 dilutions each) from the four serially diluted plasma samples was used to develop an algorithm for viral load estimation. Several strategies were evaluated to estimate low viral loads using either 1) numerical values based on extrapolated concentrations <30 copies/mL for each replicate or 2) a digital approach considering each replicate as detected or not. In the first case, measurements were modeled as i) negative binomial regression (NegBinNoCapping), ii) negative binomial regression with capping replicates with higher viral loads at 30 copies/mL if any replicate had an extrapoloated value <5 copies/mL (NegBin), iii) normal distribution (Normal), or iv) Poisson distribution (PoiQuant). In the second case, digital detection was modeled using a Poisson distribution. Because Poisson digital estimates for all positive or near all positive replicates are ill-defined, we used negative binomial regression estimates in such cases i) when all replicates were detected (PoiDigital0), ii) when zero or one replicate was not detected (PoiDigital1), or iii) when up to two replicates were not detected (PoiDigital2). The final strategy accounts for imperfect sensitivity by multiplying by 1.6 for Poisson Digital estimates (PoiDigital0Cal).

Performance metrics of each strategy were evaluated by correlation of the estimated with the nominal viral load (Table A1), calculation of the standard error of the estimated viral load (Figure A1), and calculation of the fold change of the nominal relative to the estimated viral load (Table A2). In addition, random sampling (×100) of 18 or 9 replicates was performed, and correlation and fold change metrics were compared across strategies (Figure A2).

Table A1. Correlation of the nominal and estimated viral load for each copy number estimation method

**Method**	**Pearson's correlation coefficient (r)**	**Spearman's correlation coefficient (ρ)**
NegBinNoCapping	0.81	0.61
NegBin, Normal, PoiQuant	0.92	0.97
PoiDigital2, PoiDigital1, PoiDigital0, PoiDigital0Cal	0.98	0.99

Figure A1: Standard error (SE) of the estimated viral loads (box and whiskers represent min to max, across the serial dilution range for each donor plasma sample). The SEs for PoiQuant are consistently lower than Neg Bin, which is expected since NegBin addressed the overdispersion; PoiDigital methods produced generally lower SEs than NegBin and Normal. PoiDigital0Cal has higher SE after calibration because of the multiplying factor.

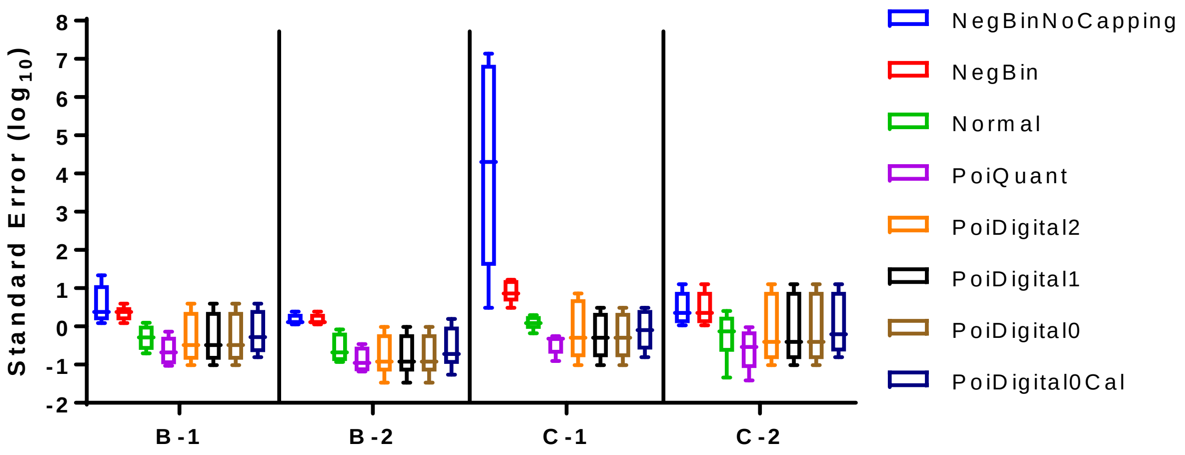

Table A2: Fold change (FC) of nominal relative to estimated viral loads for each copy number estimation method (mean, median and standard deviation) as a measurement of accuracy.

Method	Mean FC	Median FC	SD of FC
NegBinNoCapping	3.53	1.63	4.16
NegBin	2.20	1.61	1.67
PoiDigital2	1.78	1.61	0.67
PoiDigital1	1.75	1.55	0.69
PoiDigital0	1.75	1.55	0.69
PoiDigital0Cal	1.42	1.35	0.32

Figure A2 : Sampling of 9 and 18 reps (×100). Fold change and correlation of nominal relative to estimated viral loads for each copy number estimation method are shown for each random sample.

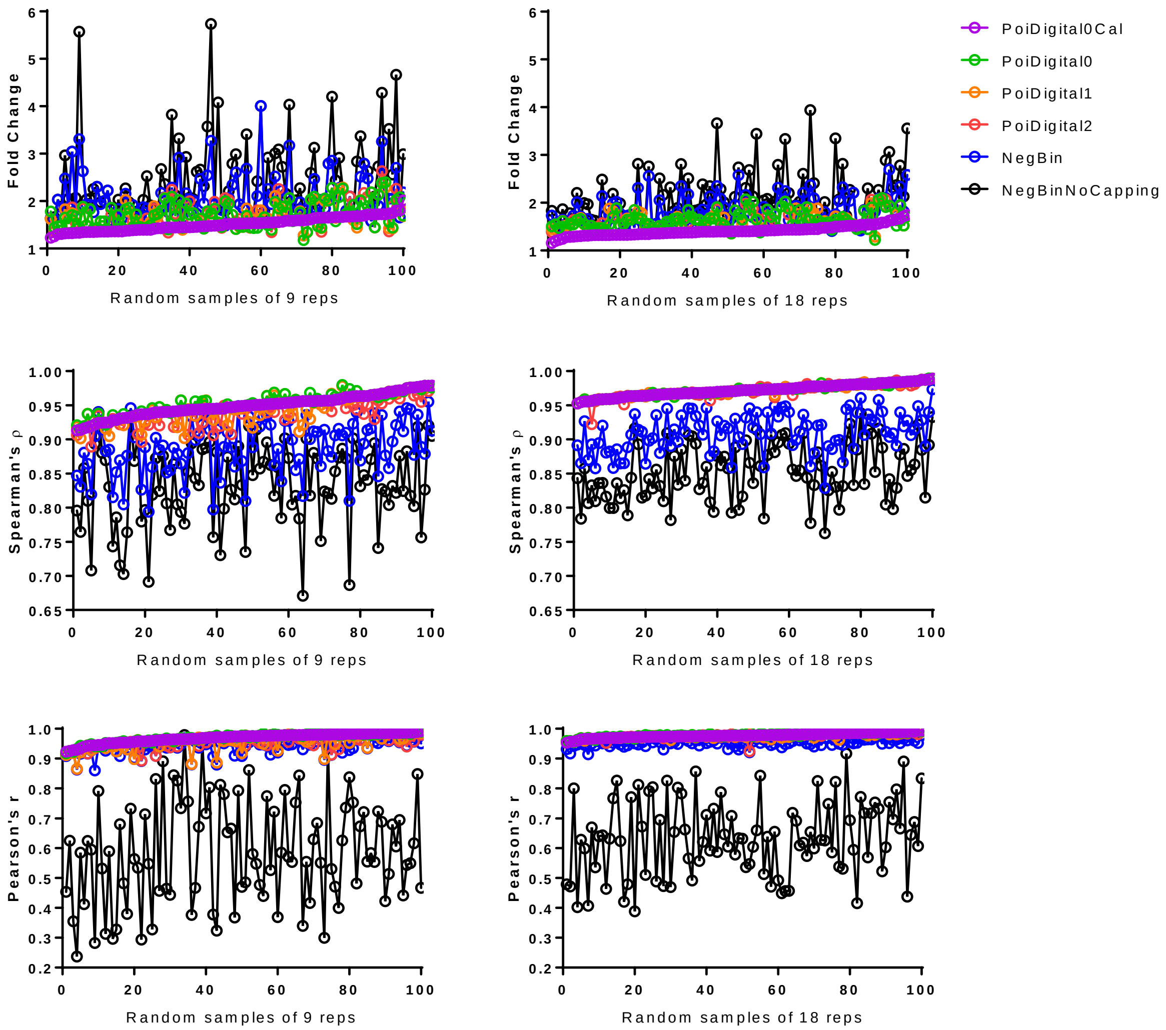

Supplement: Supplemental file 1 [file JCM.01400-20-s0001.pdf]
